# Proteomic-Coupled-Network Analysis of T877A-Androgen Receptor Interactomes Can Predict Clinical Prostate Cancer Outcomes between White (Non-Hispanic) and African-American Groups

**DOI:** 10.1371/journal.pone.0113190

**Published:** 2014-11-19

**Authors:** Naif Zaman, Paresa N. Giannopoulos, Shafinaz Chowdhury, Eric Bonneil, Pierre Thibault, Edwin Wang, Mark Trifiro, Miltiadis Paliouras

**Affiliations:** 1 Biotechnology Research Institute - National Research Council, Montréal, QC, Canada; 2 Lady Davis Institute for Medical Research, Segal Cancer Centre - Jewish General Hospital, Montréal, QC, Canada; 3 Institut de recherche en immunologie et en cancérologie, Université de Montréal, Montréal, QC, Canada; 4 Department of Medicine, Department of Oncology and Division of Experimental Medicine, McGill University, Montréal, QC, Canada; Hormel Institute, University of Minnesota, United States of America

## Abstract

The androgen receptor (AR) remains an important contributor to the neoplastic evolution of prostate cancer (CaP). CaP progression is linked to several somatic AR mutational changes that endow upon the AR dramatic gain-of-function properties. One of the most common somatic mutations identified is Thr877-to-Ala (T877A), located in the ligand-binding domain, that results in a receptor capable of promiscuous binding and activation by a variety of steroid hormones and ligands including estrogens, progestins, glucocorticoids, and several anti-androgens. In an attempt to further define somatic mutated AR gain-of-function properties, as a consequence of its promiscuous ligand binding, we undertook a proteomic/network analysis approach to characterize the protein interactome of the mutant T877A-AR in LNCaP cells under eight different ligand-specific treatments (dihydrotestosterone, mibolerone, R1881, testosterone, estradiol, progesterone, dexamethasone, and cyproterone acetate). In extending the analysis of our multi-ligand complexes of the mutant T877A-AR we observed significant enrichment of specific complexes between normal and primary prostatic tumors, which were furthermore correlated with known clinical outcomes. Further analysis of certain mutant T877A-AR complexes showed specific population preferences distinguishing primary prostatic disease between white (non-Hispanic) vs. African-American males. Moreover, these cancer-related AR-protein complexes demonstrated predictive survival outcomes specific to CaP, and not for breast, lung, lymphoma or medulloblastoma cancers. Our study, by coupling data generated by our proteomics to network analysis of clinical samples, has helped to define real and novel biological pathways in complicated gain-of-function AR complex systems.

## Introduction

Significant advances in genomic sequencing methodology have allowed a better assessment of the extent of somatic mutations accrued in common neoplasms [1], [2]. More important is the realization that tumors significantly vary genetically from one patient to another and within a singular patient there exists extensive inter-tumoral heterogeneity and intra-tumoral heterogeneity [3], [4], [5]. A significant number of these genetic alterations are missense mutations that provoke new gain-of-function properties that render a particular gene proactive to tumoral evolution and are referred to as driver mutations. A better understanding of these new properties would lead to a better interpretation of oncogenesis, but this is difficult due to a large number of different mutations, the unpredictable nature of gain-of-function properties associated with somatic mutations, the possible extensive interplay of different somatic mutants and the ensuing selection processes initiated by the microenvironment or by therapy itself. Such complex “systems” require a more global “omics” approach and more network analysis, rather than the classical single gene approach, to garner more critical information related to neoplastic evolution.

In keeping with the newly defined mutational landscape of tumors, prostate cancer (CaP) also has extensive genetic alterations that range from single missense mutations, copy number variation, splicing variants, genetic rearrangements and short DNA alterations in a large number of genes [1], [2], [6], [7], including the androgen receptor (*AR)* gene. It is not unexpected that AR mutations can add to the protein’s repertoire of powerful new functions [8], [9] and these gain-of-function attributes may allow the AR to function in an aberrant manner. A number of somatic CaP AR mutants, especially the most commonly occurring CaP AR mutation, Thr877Ala (T877A), have unique gain-of-function properties: they can bind several classes of steroids promiscuously (e.g. estrogens, progestins, glucocorticoids) with subsequent transactivation, or be hyperactivated by normal ligands [10]. Classic anti-androgen treatments [e.g. flutamide, cyproterone acetate (CPA) or bicalutamide] have generated, through selection pressure, specific somatic AR mutations, e.g. Trp741Cys (W741C) and His874Tyr, resulting in subversive ARs that are fully active with these drugs [11]. Even the next generation of anti-androgen drugs exemplified by enzalutamide (MDV-3100) has provoked specific AR mutations [12], [13]. This observation also correlates with a dramatic fall in PSA levels subsequent to anti-androgen withdrawal [11]. The T877A-AR mutations, which is also present in prostate cancer cell line LNCaP, has been reported by various individuals to occur in 25 to 33% of androgen-independent or castrate-resistant tumors [11], [14], [15], [16].

Recently, our own work strongly suggests that the AR function extends beyond its classical role as a transcription factor and includes the novel properties of RNA splicing, DNA methylation, proteasomal interaction and protein translation at the polyribosomes themselves [17]. Furthermore, the great functional diversity of the components of AR complexes exemplifies the intricate nature of protein-protein interactions associated with generating the appropriate AR biological output. These novel AR functions may mediate cellular processes and offer new areas in which somatic AR CaP mutants might “indulge” and promote CaP oncogenesis.

In an attempt to describe novel gain-of-function properties associated with mutant CaP ARs, a proteomic-coupled network analysis was performed. Multiple proteomics-mass spectroscopy investigations were carried out in order to fully characterize the protein composition of T877A-AR “complexes” (interactome) under different classes of hormone/ligand conditions reflecting the promiscuity of ligand binding associated with T877A. Critically, mutant CaP ARs may have their own unique ability to undergo and define new interactions. The coupling of the data generated by our proteomics screen to system biology analysis has been helpful in defining real and novel biological endpoints in AR complex systems, within a clinical disease perspective.

## Materials and Methods

### Cell lines

The LNCaP prostate cancer cell lines was obtained from the American Type Culture Collection (ATCC), Rockville MD.

### Cell culture, steroid hormones, ligands and stimulation experiments

LNCaP cell line was cultured in RPMI 1640 media supplemented with FBS (10%), at 37°C, 5% CO_2_ in T-75 plastic culture flasks. Once confluent, the medium was changed to RMPI supplemented with 10% charcoal–dextran stripped FBS and incubated for an additional 24 h. The following day, the medium was changed to fresh RMPI/charcoal–dextran stripped FBS for overnight hormone/ligand stimulation studies for an 18 hour period. Steroids hormones were used at the following final concentrations, 10 nM dihydrotestosterone (DHT), 10 nM mibolerone (MB), 10 nM R1881, 10 nM testosterone+10 µM finasteride, 10 nM 17β-estradiol, 10 nM progesterone, 10 nM dexamethasone, and 100 nM cyproterone acetate (CPA).

### Affinity purification and Western blotting

LNCaP whole cell lysates were prepared by freeze-thaw method with 1X PDG buffer containing the appropriate hormone/ligand [18]. Lysates were then carried over for α-AR co-immunoprecipitations [AR(N20), Santa Cruz Biotechnology, Santa Cruz, CA] overnight at 4°C. A 50% Protein A Sepharose slurry was added to each sample and incubated at room temperature for 90 min. Beads were washed three times with wash buffer (50 mM Tris-HCl pH 8.0, 150 mM NaCl, 1% Tween 20) and resuspended in 100 µL 1X SDS gel loading buffer. Samples were denatured by boiling, and resolved on a 10% SDS-polyacrylamide gel before silver staining according to manufacturer’s guidelines (BioRad) or transfer to a nitrocellulose membrane for Western blot analysis using monoclonal antibody AR(441) (NeoMarkers, Fremont, CA).

### Mass spectrometry and peptide comparison

TCEP (tris(2-carboxyethyl)phosphine) was added to the protein samples to reach the concentration of 5 mM. Samples were incubated at 37°C for 30 min. One µg of trypsin was added and the samples digested overnight at 37°C, then dried down in a SpeedVac and resolubilized in 50 µl of ACN 5%/formic acid (FA) 0.2%.

All MS analyses were performed using an LTQ-Orbitrap hybrid mass spectrometer with a nanoelectrospray ion source (ThermoFisher, San Jose, CA) coupled with an Eksigent nano-LC 2D pump (Dublin, CA) equipped with a Finnigan AS autosampler (Thermo Fisher, San Jose, CA). Twenty µl of each sample was injected on a C18 precolumn (0.3 mm i.d.×5 mm) and samples separated on a C18 analytical column (150 µm i.d.×100 mm) using an Eksigent nanoLC-2D system. A 76-min gradient from (A/B) 10–60% (A: formic acid 0.2%, B: acetonitrile/0.2% formic acid) was used to elute peptides with a flow rate set at 600 nL/min. The conventional MS spectra (survey scan) were acquired in profile mode at a resolution of 60,000 at m/z 400. Each full MS spectrum was followed by three MS/MS spectra (four scan events), where the three most abundant multiply charged ions were selected for MS/MS sequencing. Tandem MS experiments were performed using collision-induced dissociation in the linear ion trap.

The comparisons of peptide abundance across the different experimental paradigms were achieved using label-free quantitative proteomics [19], [20]. Briefly, raw data files from the Xcalibur software was converted into peptide map files representing all ions according to their corresponding m/z values, retention time, intensity and charge state. Peptide abundance was then assessed using the “peak top” intensity values. Intensities of peptides eluting across several fractions were summed together, and only a coefficient of variance (CV) allowing the maximal ion transmission was considered to calculate peptide intensity. Clustering of peptide maps across different sample sets was performed on the peptide-associated Mascot entry using hierarchical clustering with specific tolerances (+/−15 ppm of peptide mass and +/−1 min of peptide retention time). Normalization of retention time was performed on the initial peptide cluster using a dynamic and nonlinear correction that confines the retention time distribution to less than 0.1 min on average. Reproducibility changes in abundance across conditions was determined using a two-tail homoscedastic *t*-test on sample replicates to identify peptide clusters with *p*-values <0.1 with fold changes greater than 7 standard deviations. Peptide clusters fulfilling these selection criteria was inspected manually to validate identification and changes in abundance. Expression analyses were performed on proteins identified by at least two different peptide sequences. Expression values and relative standard deviation were gained by averaging the intensity differences and standard deviations of the four most intense peptide triplets after removing outlying peptide clusters. Normalized proteomic data can be found in **Table S1**.

### Datasets for network construction and gene expression analysis

All AR interactors were given NCBI gene IDs. Human protein interaction information was compiled from diverse data resources and annotation databases such as Biomolecular Interaction Network Database (BIND), the Database of Interacting Proteins (DIP), Human Protein Reference Database (HPRD), IntAct, and Molecular INTeraction database (MINT), most of which contain curated interaction data and high-throughput data. We generated a metadata of protein interactions by merging these data with our own manually curated human signaling network containing 4,000 proteins and 22,000 signaling relations [21], [22], [23].

A protein interaction network was constructed for each hormone using our manually curated human signaling network and protein-to-protein interaction network. We only considered proteins that were ≥2.5 times the hormone condition abundance (signal) vs. the vehicle control abundance from the MS dataset, to be considered significantly present. In the network, a node and link represent the protein and interaction, respectively. To find the highly interconnected regions of the network, for each hormone, we scored each pair of interaction based on the number of neighboring nodes they have in common. This gave us a matrix with all the scores between all the interaction pairs. Hierarchical clustering was applied to the matrix and a threshold score calculated based on the partition density allowing us to identify the highly interconnected regions (clusters) for each of the hormone networks.

Next, we used these protein clusters and identified GO-terms (http://www.geneontology.org/) that are significantly associated with each of the protein clusters (p-value<0.05, hyper-geometric). We also used Gene Set Enrichment Analysis (GSEA) defined pathways and performed GSEA if 25% of the protein cluster’s genes were in the pathway. For the pathways and GO-terms that were significant from the GSEA results (p-value<0.05), we also did survival analysis. We used genes associated with these GO-terms and performed GSEA using GSE21034 [24].

### RNA extractions and microarray analysis

LNCaP cells were stimulated with panel of ligands as described above, and total RNA was extracted using TRIZOL (Invitrogen, Carlsbad, CA). RNA samples were then processed by Quebec Genome Innovation Centre (McGill University, Montreal, Canada), for microarray analysis with Illumina Human HT-12 Expression Beadchip v4 (Illumina, San Diego, CA). Raw data was processed using R [22], [25].

Two global heatmaps were made. The first heatmap includes the most differentially expressed gene for each hormone using t-test (p-value<0.05), where each hormone is compared against all others. The second heatmap includes the top 10, 20, 50 and 100 genes with the highest variance. The t-test finds the most differentially expressed genes that are specific to each hormone, whereas the variance helps us observe genes that are differentially expressed across multiple hormones. GSEA analysis was performed using GSE21034 [24], of the 10, 20, 50 and 100 genes that showed the highest variance.

### Progression and Survival Analysis

Gene expression profiles, patient survival data, and demographic information for the 267 clinical prostate samples (29 normal, 181 primary and 37 metastatic tumors) were obtained from GSE21034 [24]. Breast, lung, lymphoma and medulloblastoma datasets were obtained from the Broad Institute (http://www.broadinstitute.org/cgi-bin/cancer/datasets.cgi) [26]. We examined the post-radical prostatectomy prognostic values of a subnetwork based on gene expression profiles of primary tumors, and performed Kaplan-Meier analysis by implementing the Cox-Mantel log-rank test using R as described previously [22], [25]. If the p-value is less than 0.05, the subnetwork was treated as statistically significant to classify the tumors into non-metastatic and metastatic tumors. We stratified recurrent vs. non-recurrent CaP cancer based on the following criteria: PSA (≥4 ng/mL), Gleason (≥7), Tumor Stage (≥3) and combined (PSA+Gleason+Tumor).

### Structure Preparation for MD simulation

The Crystal structure of DHT (1I38) and CPA (2OZ7) bound to T877A mutant AR-LBD and testosterone (2AM9) and R1881 (1E3K) bound to wild type AR-LBD are available in the Protein Data Bank (PDB). The complexes of different ligands bound to the T877A mutant AR-LBDs were further prepared for MD simulations using Xleap [27] in AMBER10 [28]. The generalized AMBER force field (GAFF) and ff99SB [29] parameters were used for eight different ligands. The complex was solvated in a truncated octahedron TIP3P [30] water box. The distance between the wall of the box and the closest atom of the solute was 12.0 Å, and the closest distance between the solute and solvent atoms was 0.8 Å. Counterions (Cl^−^) were added to maintain electroneutrality of the system. Each system was minimized, first by applying harmonic restraints with force constants of 10 kcal/mol/Å^2^ to all solute atoms; second, by heating from 100 to 300 K over 25 ps in the canonical ensemble (NVT); and lastly by equilibrating to adjust the solvent density under 1 atm pressure over 25 ps in the isothermal–isobaric ensemble (NPT) simulation. The harmonic restraints were then gradually reduced to zero with four rounds of 25-ps NPT simulations. After additional 25-ps simulation, a 15-ns production run was obtained with snapshots collected every 1 ps. For all simulations, 2 fs time step and 9 Å non-bonded cut-off were used. The particle mesh Ewald method [31] was used to treat long-range electrostatics and bond lengths involving bonds to hydrogen atoms were constrained by SHAKE [32].

## Results

### Comparative network characterization of T877A-AR complexes: interactome and gene expression studies

Our ability to capture both ligand-bound and unliganded full-length wild-type AR by affinity chromatography under physiological conditions has previously allowed us to pursue a proteomics approach in order to characterize the components of wild-type AR complexes. This was done by subjecting such complexes to tryptic digestion followed by mass spectrometry (MS) to assign protein identification, in effect creating AR interactomes initiated by ligand binding [33]. To our MS data, a label-free quantitative method has now been applied across the different experimental paradigms (see Materials and Methods) [19], [20], which allowed us to obtain data related to protein identification, along with abundance, thus allowing for direct comparisons between stimulation conditions.

The aim of differential hormone stimulation conditions will allow us to determine whether disease etiology of the T877A-AR mutation is dependent upon ligand and co-factor status. Therefore, we used LNCaP cells that endogenously express the T877A-AR mutation to characterize the ligand promiscuous protein interactome complexes under different hormonal conditions, in order to highlight the possible distinct complexes that may be linked to disease progression. LNCaP cells were stimulated with the following hormones, four androgens: DHT, mibolerone (MB), R1881, or testosterone (in the presence of finasteride (to prevent the conversion of testosterone to DHT), or 17β-Estradiol, progesterone, dexamethasone, or the anti-androgen cyproterone actetate (CPA), alone or with no-ligand/alcohol-vehicle control. Hormone stimulated T877A-AR complexes were immunopurified with an N-terminal specific AR antibody, which would not interfere with hormone binding to the AR ligand-binding domain. Eluates from all experimental conditions were analyzed by LC-MS/MS. In order to increase our sample frequency for peptide detection in our MS analysis, each experimental condition was performed four times. Our proteomics data is a compilation of only fully characterized proteins, with full gene ontology and function.

**Figure 1:**
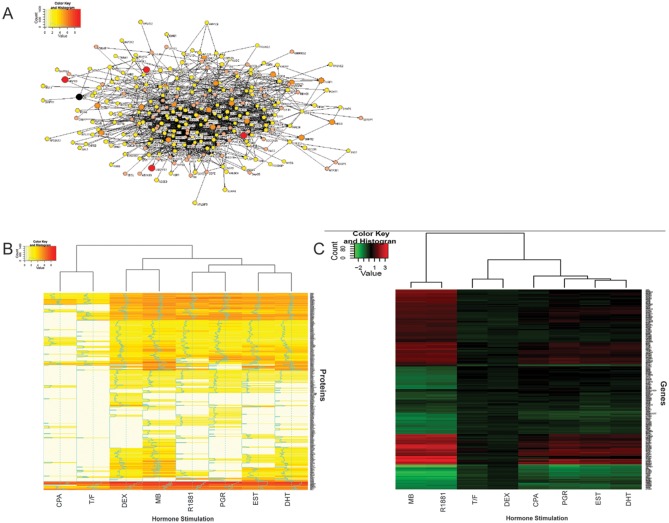
Characterization of T877A-AR protein interaction and gene expression profiles. **A.** Quantified protein interaction network of DHT stimulated LNCaP cells. The value of each protein, defined by our label-free quantitative MS, is distinguished by both color and size, and sub-sequent protein-protein interactions. The AR is designated by the black circle. To determine the relationship between protein interaction and gene expression patterns, hierarchical clustering of multi-panel hormone-stimulated LNCaP cells was carried out. **B.** Interacting proteins identified by mass spectrometry. **C.** Most variably expressed genes upon ligand stimulation. Although it has been suggested that all hormones used are able to activate androgen-dependent gene transcription with the T877A-AR mutant receptor, there are differences between AR protein complexes and AR gene expression patterns. This cluster analysis illustrates that even the synthetic androgens like Mibolerone (MB) and R1881, have similar gene expression profiles, their protein-interaction complexes are more similar to dexamethasone (DEX) and progesterone (PGR), respectively, than to either natural androgens testosterone and DHT. Moreover, even natural androgens (DHT and testosterone) can be segregated by their protein complexes. This would suggest that there are functions for the AR beyond gene transactivation. Protein and gene expression values are given as a ratio of quantified protein of each stimulation vs. vehicle control stimulation.

Quantitative MS data, for each of the eight hormone stimulation conditions, was used to create a protein interaction map (**Figure 1A**). The protein interaction network map, allows for a visual analysis of the relationship of the interaction of each protein with the mutant AR. It is most likely that not all proteins interact directly with the mutant AR, but can through intermediate proteins. Further ontological function classification (see Materials and Methods) is based on this interaction network map, by discerning significant clusters of interacting proteins based on the number of protein-protein interaction connections. Of the eight hormone stimulation T877A-AR protein lists, we then systemically applied hierarchal clustering analysis to the experimental conditions. Hierarchical clustering heat-maps representing the grouping of between whole T877A-AR agonist and antagonist experimental treatments were then generated (**Figure 1B**). Between the different experimental conditions (hormone treatments), a comparative network analysis was applied [21], [22], [23], and although four different androgens were used (DHT, testosterone, MB and R1881), the proteomic profiles of these androgen ligands do not segregate together, and we observed that progesterone and dexamethasone AR complexes have proteomic profiles that look like R1881 and MB, respectively. Moreover, the protein interaction complex for AR-estradiol-stimulated complexes was most similar to the AR-DHT response interactome.

We also proceeded to characterize the gene expression patterns of the multi-panel hormone stimulated LNCaP cells. Analysis of most variably expressed genes between the hormone conditions gave a hierarchical clustering pattern that was much different to the T877A-AR protein-interaction profile (**Figure 1C**). Quite clearly, we again observed that the different androgens used in these stimulation profiles do not segregate together, and that synthetic androgens, like R1881 and MB, do not have the same AR-stimulated transactivation profiles as the natural ligands like testosterone and DHT. Moreover, the functional ontological properties between protein-interaction vs. gene expression profiles of our differential ligand stimulated cells also appear to be very different. Discerning the impact on disease progression from these profiles was of particular interest.

To establish statistically significant biological functions, we implemented the incorporation of Gene Ontological (GO)/pathways terms using DAVID (Database for Annotation, Visualization and Integrated Discovery, http://david.abcc.ncifcrf.gov/). Using the protein interaction data from all the ligand stimulation conditions, the major ontological functions are: RNA pol II-dependent transcription, protein biosynthesis, with components of the translational machinery (translation initiation, elongation factors, ribosomal proteins and other regulatory proteins), RNA metabolism (specifically RNA splicing), DNA repair (through an interaction with members of the DNA repair complex), and the proteasome/ubiquitination pathways (see **Table S2**). In contrast, the ligand-dependent gene expression pattern ontological classes included pathways involved in DNA replication, steroid/sterol biosynthesis, and apoptosis (see **Table S3**). Thus, although the AR is classically described as a transcription factor, its proteome profile would suggest that the AR is capable of functions beyond what has initially been described as a gene activator.

### Structural analysis of hormone binding to the T877A-AR

**Figure 2:**
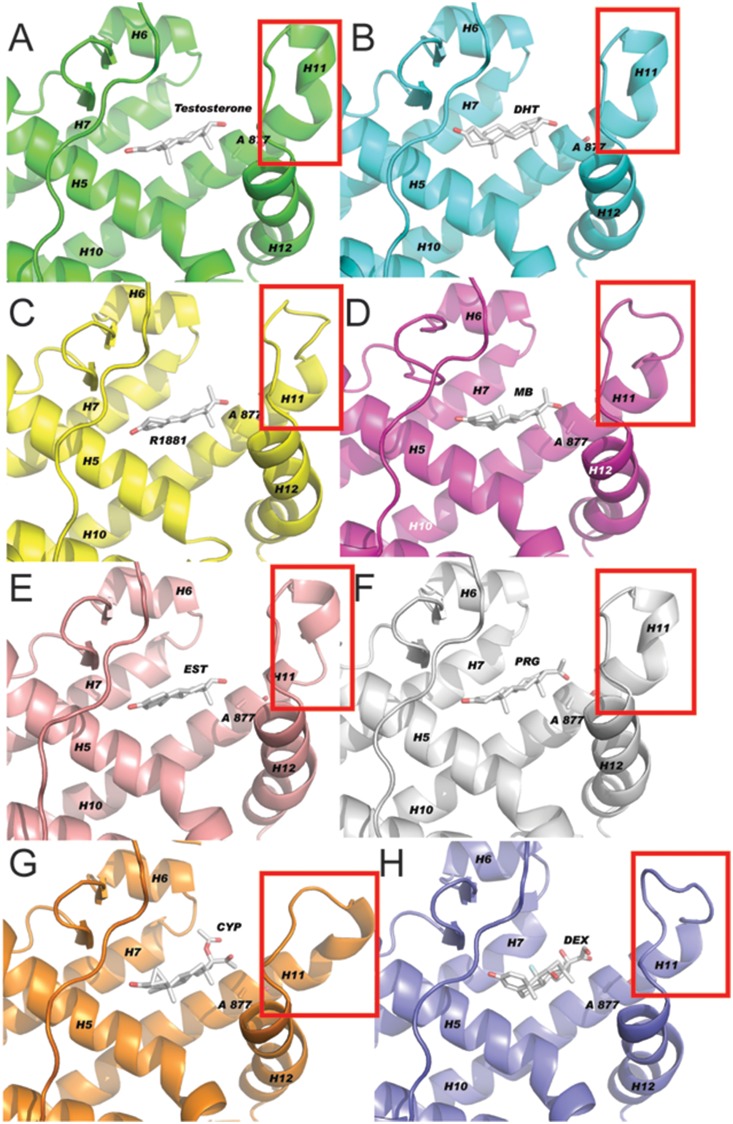
MD average structure of T877A mutant of AR ligand-binding domain with eight different ligands. Docking program WILMA was used to examine structural changes of the ligand-binding domain of the T877A mutation, upon binding to ligand binding. Illustrated is the average structure of T877A-AR mutation ligand binding domain with eight different ligands. **A.** testosterone, **B.** DHT, **C.** R1881, **D.** MB, **E.** estrogen (EST), **F.** progesterone (PGR), **G.** CPA, **H.** dexamethasone (DEX). A red box highlights the changes in the helix α 11 loop upon binding to each hormone ligand.

To explore the possible mechanisms by which ligands binding to the mutant AR create different sets of AR interacting proteins, we obtained the detailed conformation of the receptor, using 15 ns molecular dynamic (MD) simulation studies (see Materials and Methods) of the eight different ligands used. Using the docking program WILMA (**Figure 2**), we obtained structural data of the mutant AR bound with progesterone, estrogen, dexamethasone and MB. The structural data of the mutant AR with testosterone, DHT, R1881 and cyproterone acetate are already available and as such, these structures served as the starting points for the MD simulation studies.

**Figure 3:**
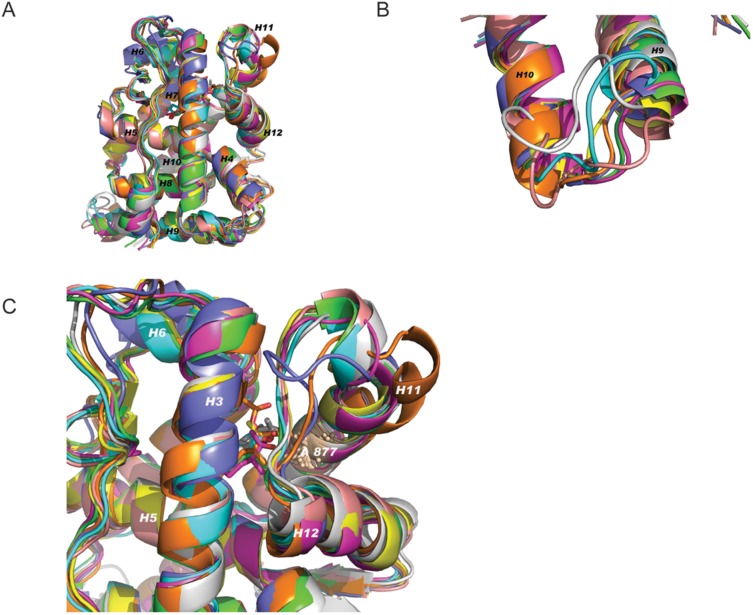
Loop structure fluctuations. **A.** Superimposed average structures of all eight ligand bound receptor complexes. **B.** Regions of T877A AR-LBD loop between Helixα9 and Helixα10 showed maximum flexibility. **C.** Expanded view of Helix α11 and Helix α12 regions of T877A AR-LBD bound to eight different ligands studied in this study. Residues of α11 and α12 and loop between them showed higher flexibility compared to other regions of the receptor where T877A is located. Color corresponds to the following ligands: Green - testosterone, Cyan- DHT, Yellow-R1881, Pink- MB, Rose- EST, Grey- PGR, Orange-CPA, Purple-DEX.

Mutant AR MD simulations were performed over 15 ns production runs. To inspect the local flexibility of each protein/hormone complex, we calculated the root-mean-squared deviation (RSMD) fluctuations of backbone atoms of each amino acid residue for each mutant AR complex. In the study of globular protein conformations, one customarily measures the similarity in the three-dimensional structure by the RMSD of the central carbon atoms in amino acids, after optimal rigid body superposition. The most significant fluctuations correspond to loop regions between α3/α4, α9, α10/α11 and α11/α12 and the helices α11 and α12 themselves of the LBD of AR (**Figure 3**). It can be seen that the loop between α9, α10 and α11 is the most flexible region in all the complexes (**Figure 3B**). Among the eight different AR ligand bound complexes, the testosterone- and estradiol-bound complexes demonstrate the highest flexibility, with some loop residues having RMSD fluctuations as high as 2.4 Å. Although these loops are distant from the hormone binding pocket, they are exposed to the surface and may serve a role as potential binding sites to other protein partners.

The other major differences observed between the mutant AR complexes are the positions of α11 and 12, which are known to be critical for dictating hormone binding and co-activator interactions. Residues of α11 and α12 and the loop between them showed higher flexibility (**Figure 3C**). The T877A-AR mutation located in α11 allows for a more spacious hormone-binding pocket and will accommodate steroids with different extensions within the D ring.

The examination of the nature and size of the solvent accessible surface area (SASA) of proteins is an important tool to measure potential interaction propensity with neighboring proteins. We calculated the average SASA of each AR complex from the 15 ns MD trajectory. No large differences were observed among the calculated SASAs of different AR mutant complexes, which ranged from 12,124 to 12,390 Å^2^. These results show that the differences are mostly associated with the α11–α12 regions of the AR-LBD, where the T877A mutation is located. Therefore, we decided to compare the dynamics among the eight different complexes, specifically in this region, by using RMSD matrices (**Table 1**). The matrices, which essentially capture extreme movements, reveal regions of high flexibility, especially for CPA and dexamethasone, compared to other AR-ligand complexes.

**Table 1:** Two dimensional RMSD matrices of the residues of Helix α11 and Helix α12 and loop between them without fitting.

	DHT	TEST	MB	R1881	CPA	DEX	EST	PRG
**DHT**	0	1.72	1.83	1.79	3.11	3.23	1.79	2.03
**TEST**	1.72	0	1.69	2.05	3.17	3.19	1.73	2.2
**MB**	1.83	1.69	0	2.19	2.93	2.93	1.88	2.26
**R1881**	1.79	2.05	2.19	0	3.54	3.38	1.98	2.11
**CPA**	3.11	3.16	2.93	3.54	0	3.28	3.14	3.4
**DEX**	3.23	3.19	2.93	3.38	3.38	0	3.43	3.72
**EST**	1.79	1.73	1.88	1.98	3.12	3.46	0	1.82
**PRG**	2.01	2.18	2.26	2.11	3.41	3.72	1.83	0

Computational modeling has also been carried out for a number of other AR somatic mutations in the ligand-binding domain (LBD), and show sensitivity to a broad range of hormone ligands, including, AR-W741L[34], -L701H [35], [36], [37], [38], -H874Y [39], [40], [41] and -F876L [13]. Determination of the LBD structure between AR-WT and -H874Y (present in 22Rv1 cells), when bound to Testosterone in the presence of the N-terminal FXXLF motif peptide or the TIF2 coactivator peptide, found that all structures conformed to the canonical nuclear receptor LBD fold [41]. Moreover, the -H874Y DHT and R1881 structures conformed to -T877A and -W741L LBD bound to steroid and nosteroid ligands [34], [42]. The double AR-mutant cell lines MDA-PCa-2a and MDA-PCa-2b, possess the L701H and T877A somatic mutations, these cells shows similar AR transactivation and LBD structural properties to the single AR-T877A mutant LNCaP cells to a broad spectrum of steroid ligands and anti-androgens, but also show an increased sensitivity cortisol steroids [35], [36]. Most recently, the structure AR-F876L mutations has been investigated that allows the cell to use the new anti-androgen enzalutamide as an agonist. Similarly the Leu876 mutation allows for the antagonist-agonist switch, by accommodating of enzalutamide to the ligand binding pocket [39]. Although only LBD structural data is available for the AR-F876L mutant, we don’t believe that enzalutamide vs. androgen binding would significantly alter helices movement to drastically affect overall global AR structure, as has been observed with other AR LBD mutants.

### AR protein interaction functional clusters, but not clinically derived gene expression profiles, correlate with CaP progression and survival outcome

From each of the ligand mutant AR protein interaction network, we identified specific sub-network modules. These sub-networks suggest hormone-specific activated pathways involved in either tumor initiation or progression. The characterization of ontological functions across the stimulation conditions is important as these differences or similarities in interacting proteins within the T877A-AR mutant complex may account for unique or shared cellular properties contributing to disease progression and outcomes. Therefore, we annotated significant GO-terms directly on sub-network modules extracted from the eight different hormone-protein interaction networks to highlight functions that may be unique to each of stimulation condition, and extracted the list of genes corresponding to those GO-terms.

**Figure 4:**
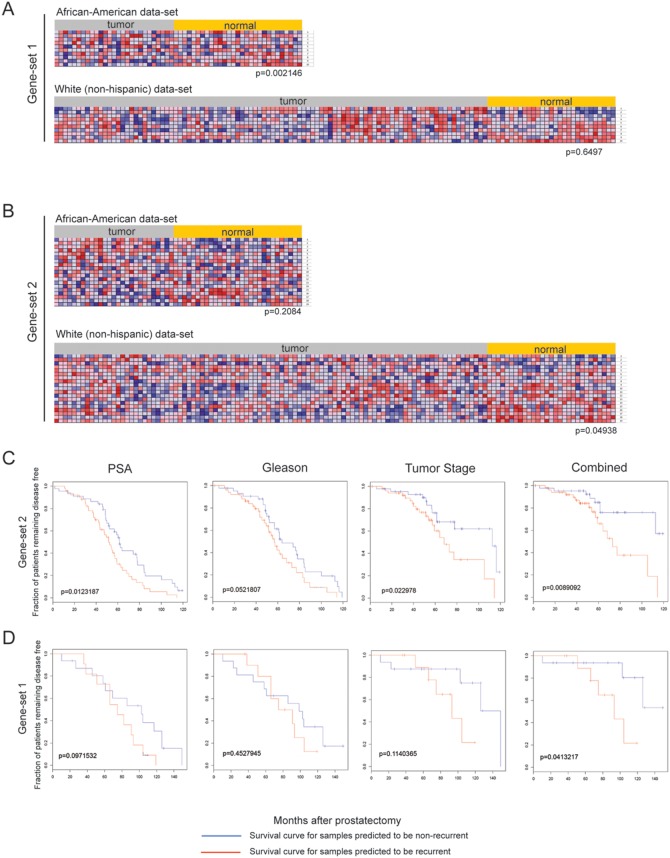
Gene expression profiles of AR protein sub-networks correlate to CaP progression and outcomes. GSEA was applied to determine the correlation of transcript expression profiles of AR subnetwork protein clusters from raw microarray data of normal and primary tumor datasets of clinical prostatic samples (GSE21034). **A.** Illustrated is an enrichment profile of a single AR subnetwork which has significant differential expression between normal and tumor datasets, and was only significant for a population of White (non-Hispanic) but not African-American men. **B.** Enrichment profile for African-American men gene-set 1. Survival outcome analysis was followed-up on these gene-sets. Disease-free survival of CaP patients stratified by serum PSA, Gleason score, Tumor stage, and combined risk (PSA, Gleason and Tumor Stage) with respect to the AR subnetwork cluster. Kaplan-Meier analysis was used to plot the fraction of at-risk patients remaining free of disease (y-axis) at the indicated time after radical prostatectomy (x-axis). Patient stratification is based on serum PSA (≥4 ng/mL), Gleason score (≥7), Tumor Stage (≥T3) and combination of serum PSA, Gleason score and Tumor Stage score values. **C.** White (non-Hispanic) functional cluster gene-set 2. **D.** African-American functional clusters gene-set 1.

Using the lists of genes from the annotated GO-terms (now to be referred to as “gene-sets”), we determined whether these gene-sets are enriched in the publicly available clinical prostatic tumor microarray dataset, by applying Gene Set Enrichment Analysis (GSEA). We used the clinical data set GSE21034 [24], containing 247 clinical specimens (29 normal, 181 primary and 37 metastatic tumors). Initial analysis of all primary tumors from this data-set did not yield obvious enrichment of any gene-sets. However, after further inspection of the data, unique features were noted in certain tumor samples. Thus, upon returning to the patient pathology information that accompanied the clinical data-set, two diverse patient populations could be immediately discerned, and thus we resegregated our data-sets between 142 White (non-Hispanic) and 25 African-American samples [other population groups (Hispanic and Asian) in the 181 available primary samples were too few to perform GSEA]. By segregating the dataset along available ethnic demographical information, we immediately were able to distinctly differentiate gene-sets between White (non-Hispanic) *vs*. African-American populations. From these results, we identified 138 T877A-AR -interacting protein sub-network modules (gene-sets) that show significant (p≤0.05) enrichment of the T877A-AR -interacting partners that discerned CaP primary tumors *vs.* normal samples. Two gene-set examples are shown in **Figure. 4**.

**Figure 4A** represents a gene-set (gene-set 1) that is significantly enriched among primary tumors of the African-American population, however subsequent GSEA of the same gene-set did not show significant enrichment in primary tumors of White (non-Hispanic) group. One of the unique features of the African-American gene-set 1 is that is enriched in normal rather than tumor samples. This suggests that these genes may have anti-tumorgenic properties or subsequent loss of the expression may contribute detrimentally to prostate disease. Of particular note, the genes represented in gene-set 1 are part of the transcription-dependent DNA repair pathway. **Figure 4B** shows another gene-set (gene-set 2) that is significantly enriched in primary tumor samples of White (non-Hispanic) males and not in the corresponding African-American cohort. Furthermore, the GSEA results of these two T877A-AR interacting protein gene-sets are also distinct, and not shared, between primary tumor data of population groups of White (non-Hispanic) or African-American males. However, African-American gene-set 1 was significantly enriched in metastatic tumor data of White (non-Hispanic) males (data not shown). Due to limited data available on metastatic tumors from the African-American demographic (2 data sets), and reciprocal analysis could not be performed. This data highlights that we can identify gene-sets that show population-specific distinctions from primary tumors, but also suggests that the molecular characteristics of AR function underlying disease etiology in metastatic tumors may be common between African-American and White (non-Hispanic) men.

We next analyzed whether or not these distinct population T877A-AR gene-sets were predictive of 10-year survival outcomes, available from the GSE21034 data set [24], based on the following criteria; PSA (≥4 ng/mL), Gleason (≥7), Tumor stage (≥T3) or Combined (PSA + Gleason + Tumor stage). From our initial 138 characterized gene-sets, we identified 10 AR-interacting protein complexes that are indeed predictive for disease outcomes, 8 representing the White (non-Hispanic) population and the remaining the African-American group. From these 10 gene-sets (see **Table S4**), a single gene-set from the White (non-Hispanic) population (gene-set 2) was able to predict disease outcomes across all scoring criteria, (PSA, Gleason, Tumor Stage, and combined (**Figure 4C**). This particular gene set did not exhibit any predictive value when analyzed against the African-American cohort of samples. The African-American gene-set (gene-set 1) was only able to predict disease outcome using the combined criteria (PSA+Gleason+Tumor Stage) (**Figure 4D**). All other gene-sets, from all population groups, were able to predict survival outcomes for only one scoring criteria (**see Figure S1**). Furthermore, these predictive clusters were shared between all the hormones, supporting the results from our dynamic modeling study, where the T877A mutation accommodates all steroid hormones to and exhibits very subtle structural differences, although the overall structures appear to elicit the same functional interaction platform.

A similar analysis to that performed with our proteomic data was performed using our LNCaP multi-panel hormone microarray gene expression data across our hormone stimulation conditions. We selected 10, 20, 50 and 100 of the most variably expressed genes from our microarray data set to assess the ability to predict disease progression and outcome between White (non-Hispanic) or African-American men. We identified two gene-sets of 10 and 50 genes respectively that were able to distinguish between normal *vs*. tumor in White (non-Hispanic) men, but not African-American men. There appears to be no predictive value associated with each different class of hormone stimulation, irrespective of whether the hormones act through T877A-AR or through their cognate receptor. It is also apparently clear that the two different data-sets (protein interactome *vs*. gene expression), result in two different capabilities of predicting disease outcomes. This is a direct result of linking ontological function to a specific protein sub-network vs. arbitrarily selecting a defined number of genes linked solely to expression profiles.

Finally, although LNCaP cells are derived from a metastatic CaP lymph-node biopsy from a White (non-Hispanic) male, other cell lines also possessing the T877A-AR mutation, MDA-PCa-2a and MDA-PCa-2b, from bone metastatic CaP from African-American also exist. However, extensive genome-wide gene expression characterization between these cell lines and LNCaP, have found them to be most similar to one another vs. other androgen sensitive cell lines, LAPC4 (possessing a wild-type AR) or 22Rv1 (H874Y-AR) or vs. AR-null cell lines PC3 and DU145 [43]. Thus, if this differential hormone stimulation experiment were to be performed using MDA-PCa-2a or -2b cell lines, we would identify the same AR protein complexes *in vitro*, as LNCaP, and would also predict disease survival *in vivo* from clinical population data.

### Characterized T877A-AR protein gene-sets cannot predict survival outcomes in four other non-CaP cancers

**Figure 5:**
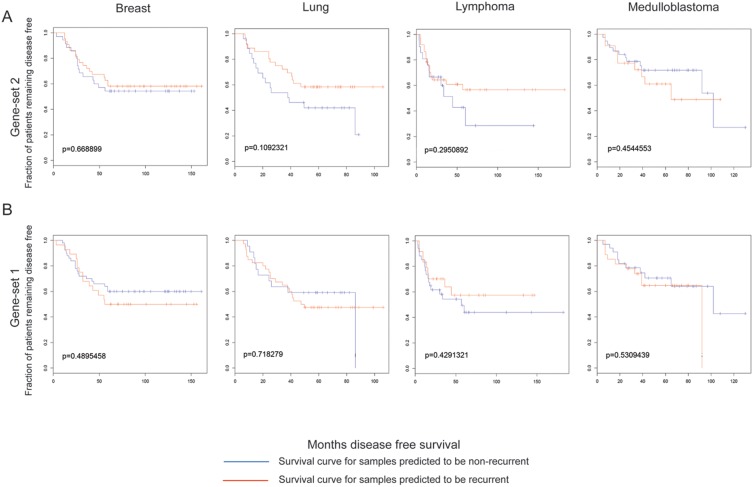
Disease-free survival of 4 non-CaP cancers. Datasets were retrieved from the Broad Institute (http://www.broadinstitute.org/cgi-bin/cancer/datasets.cgi) representing expression and disease outcomes for the following cancers: breast, lymphoma, lung and medulloblastoma, to determine survival outcomes for the gene-sets 1 and 2, as described in Figure 4.

We subsequently determined whether these predictive gene-sets were cancer specific and extracted gene expression datasets with available clinical outcome profiles for breast, lymphoma, lung and medulloblastoma clinical samples [26]. The gene-sets illustrated in **Figure 4** did not give significant outcome values for any of the four other cancers (**Figure 5**). Furthermore, the remaining 8 gene-sets, described above, also lacked significant predictive outcomes for the same 4 non-prostate cancers analyzed (see **Figure S2**). In hormone-dependent breast cancer, certain ethnic population differences have been observed, with higher incidences of breast cancer occurring in African-American woman vs. other groups [44], [45], [46], but similar population demographic data used for the CaP cohort analyzed within our study was not available for the non-CaP cancers used in this analysis. Such genetic expression data would have been useful for further confirmatory follow-up studies. However, the expression of the AR has been described in a number of non-CaP cancers, especially breast cancer [47], [48], [49], [50], [51], [52], [53]. It has also been shown that several cell lines from these non-CaP cancers show androgen sensitivity and androgen-dependent gene expression profiles similar to CaP cell lines. However, we have identified AR interactome gene-sets that can that can differentiate between CaP and non-CaP disease survival which suggests that there are unique molecular characteristics of AR function in CaP and part of a CaP-specific pathway in neoplastic development, and also that these gene-sets can be used to predict CaP disease outcomes between genetically diverse groups.

### White (non-Hispanic) gene-set can predict CaP disease outcome based on gene copy number

**Figure 6:**
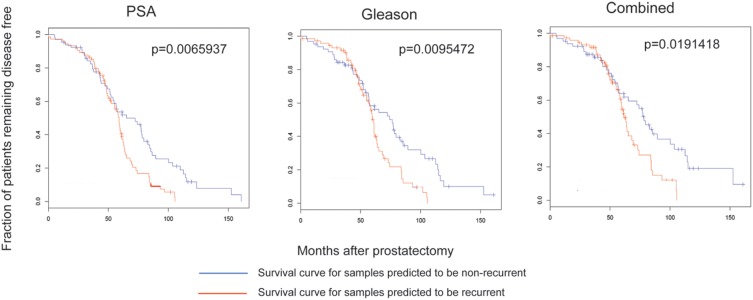
Survival outcomes reflected by Copy Number Variation. Survival outcomes based on copy number variation (GSE21035) of gene-set 2 and same patients described in Figure 4, was applied. Patient stratification is based on serum PSA (≥4 ng/mL) and Gleason score (≥7) and combination of serum PSA and Gleason score values.

Finally, to deduce a molecular mechanism to account for differential gene expression patterns for each gene-set, we analyzed copy number variation of a CGH array dataset (GSE21035) [24], for the patients used for our GSEA and survival prediction outcome. For the White (non-Hispanic) gene-set 2, we were able to confirm that patient survival based on the criteria of PSA value, Gleason score and combined (PSA+Gleason), was dependent on copy number variation (**Figure 6**). This was the only gene-set that was predictive for survival outcomes based on copy number variation.

## Discussion

Knowledge of various molecular mechanisms of action contribute to our understanding of wild type AR function. Most mechanisms require the involvement of ligand binding and interacting partners. Examples of this include AR interactors involved in gene transactivation, including HSP70, HSP90, p300 and components of the RNA polII complex [54]. Gain-of-function somatic mutations, abundant in cancerous tissues, typically add new functions, adding to the complexity of physiological and disease outcomes. We investigated the T877A-AR mutation, as it represents the most common AR mutation in clinical CaP specimens, and is the AR mutation found in the most studied prostatic cancer cell line, LNCaP. Mutations like T877A-AR, and several others in the ligand-binding domain of the receptor, allow the AR to bind to other classes of steroid ligands such as estradiol, dexamethasone and progesterone, including anti-androgens such as CPA, resulting in subsequent AR dependent gene transactivation [55]. It is also now clear that as cancers evolve through many somatic mutations [56], [57] and undergo selection processes induced by classical drug therapies themselves. Futhrermore, a number of studies have used LNCaP cells as a model for studying the progression from androgen-dependent to –independent/castrate resistant prostate cancer (AIPC/CRPC) state and support the hypothesis that continuous AR activity and signaling continues to be one of the most important mechanisms in CRPC [58], [59], [60], [61], [62]. These studies have substantiated extensive genetic alterations that range from single missense mutations, to copy number variations, splicing variants, genetic rearrangements and short DNA alterations in a large number of genes and AR co-factor interactions to reproduce androgen-independent scenario [1], [2], [6], [7], [36], [63], [64]. Modeling AIPC using LNCaP cell lines and actual tissue from AIPC patients, Wang et al., 2009 [64] found that the of gene expression regulated by the AR in the absence of hormone is distinct from androgen-regulated program and can selectively and directly upregulate M-phase genes found in androgen-independent CaP and may explain why maximal androgen deprivation (AR antagonists and LHRH inhibitors), and cannot prolong androgen-independent survival. Most recently, it was found that overexpression of AR was a result to prolonged exposure of LNCaP-derived xenografts with the anti-androgen enzalutamide, and was similar to chronic androgen depletion, as a significant mechanism for drug resistance and CRPC development [65].

In this study we characterized whole mutant AR protein complexes with several classes of steroids and ligands known to bind T877A-AR. The specific interactomes were dependent on the ligand utilized; so too were the specific gene expression profiles associated with each ligand. Thus mutant AR gain-of-function properties are not singular but multiple, dictated by the class of steroid hormones used. Further exploration of other adrenal androgens such as DHEA or androstenedione or other anti-androgens such as flutamide and bicultamide were not examined in this study, however, we did select a diverse class of ligands known to bind to the T877A-AR variant.

High-throughput gene expression microarray approaches described in CaP cells have identified hundreds of androgen-regulated genes and also characterized genome-wide AR recruitment sites [66], [67], [68], [69]. The classical AR complex contains general transcription factors, coregulators and specific transcription factors that associate either directly or indirectly with the AR to enhance or repress its transcriptional activity function without themselves necessarily binding to DNA. As shown in our recent proteomic studies [17], including this one, AR complexes may also include a larger number of functionally diverse proteins involved in a multitude of “non-classical” AR cellular processes such as histone acetylation, DNA methylation, ubiquitination, RNA splicing, apoptosis, and protein synthesis, with all pathways found to be dependent on hormone stimulation conditions.

From our data, there are several clusters of AR-interacting proteins that are worth exploring to understand their role in disease progression. The first cluster of AR-interacting proteins is unique to the African-American population group and consists of the following proteins: **ERCC1, ERCC2, ERCC3, ERCC5** and **FEN1**. The function of these proteins is required for mediating DNA damage excision repair. However, they are known components of the RNA polymerase II transcriptional complex [70] and ERCC2 and ERCC3 have been previously published as AR interactors [71]. Experimental evidence show that over-activity of this complex can lead to instability of CAG/CTG triplet repeats, resulting in a shortening of the repeat [72], [73].

The second cluster of AR-interacting proteins is unique for the White (non-Hispanic) population and is involved in chromatin remodeling and histone deacetylation activity. From this cluster we identified the following proteins: **KDM1A, KDM4C, SIRT1, CTBP1, NR2C1** and **SMARCD3**. NR2C1 has been previously described as an AR interactor [74]. The selection of this cluster of proteins for further analysis would be interesting because of the increased attention histone deacetylase inhibitors that are garnering in cancer biology [75].

A final pathway for further investigation, and unique to the White (non-Hispanic) group of men, is the role of the AR in participating in the negative regulation of apoptosis via its interaction with **BCL2, RELA, FAS, EEF1A2** and **NR4A2**. The role of these proteins have been well described in apoptotic pathways, and RELA (NFκB p65) is a well characterized interactor of AR [76] and BCL2 [77]. A proposed mechanism by which these proteins may facilitate regulating apoptosis would be via a signal transduction cascade that would negatively regulate pro-apoptotic genes and proteins [77].

Using the T877A-AR hormone-specific interaction complexes as a basis for a novel systems biology network analysis exercise established gene-sets with clear predictive CaP clinical outcome value. In doing so, we confirmed the critical importance of the genetic backgrounds of the CaP individuals in the clinical dataset. Without segregating the microarray expression data of CaP patients along White (non-Hispanic) and African-American datasets, no defined gene-sets with predictive clinical values could be identified. Once the data had been segregated, gene-sets with very powerful clinical outcome parameters were discovered. African–American men have long been considered to have clinically different CaP from White (non-Hispanic) men, based on their genetic background [78], [79]. This is not entirely surprising, as African–American descent has long been associated with higher incidence and more aggressive disease, characterized by greater tumor volume for each clinical stage, have greater PSA levels and a more aggressive cancer for Gleason score of 8 or greater with compare to White (non-Hispanics) males [80], [81]. Investigations, excluding socio-economic disparities that would limit individuals to health care [82], to explain racial differences between African-American vs. White (non-Hispanic) males, have excluded hormones levels, as serum testosterone levels (later in life) have similar levels at the time of prostate biopsy and in the their prostate biopsy tissue [83], [84]. However, analysis of AR expression in malignant vs. benign prostate tissue African-American males found to be 27% more likely to stain positive for AR and the nuclear localization of the AR was 81% greater than White (non-Hispanics) [85]. It was also obersved that there was significantly expression of CaP biomarkers that those of white men, one of which was the AR [86]. Interestingly, African-American males vs. other ethnic groups display shorter *AR* CAG repeat lengths which code for the polyglutamine tract of the AR [87]. AR with shorter polyglutamine tracts exhibits higher AR activity and represents a potential risk factor for CaP [88], [89], [90]. Therefore, dysregulation of DNA repair function, specifically loss of expression, and the link to contraction of CAG tract length in African-American males is a mechanism to investigate as a mechanism to explain racial differences in CaP. Furthermore, to gain more insight into CaP molecular/genetic etiology, our approach, encompassing proteomics, expression studies and network analysis, would benefit from investigating even more populations of diverse genetic origin backgrounds that have very low rates of CaP including Chinese and Middle Eastern men. By identifying such protective pathways of AR function, along with identifying powerful prognostic tools to predict disease, now we can assess pathways to also offer novel therapeutic targets.

In conclusion, our unique approach of using an important gain-of-function AR mutation, has generated gene-sets along functional organization lines showing that we can distinguish prostatic disease between White (non-Hispanic) and African-American men. Moreover, in our study for classification of AR function based on interaction profiles was a much more powerful tool predicting disease and survival outcomes than analyzing androgen-dependent gene expression patterns. The identification of these new functional properties of the AR and somatic mutations of the AR, further suggests that there is a role for the AR beyond that as a transcription factor and also implicates the ability of non-androgenic hormones to activate CaP disease-linked pathways.

## Supporting Information

Figure S1 **CaP 10-year survival outcomes forT877A-AR Gene-sets 3–10.**
(PDF)

Figure S2 **Disease-free survival outcomes of 4 non-CaP cancers for Gene-sets 3–10.**
(PDF)

Table S1 **Normalized Proteomic Data.**
(XLS)

Table S2 **GO-Term definitions of Proteomic data.**
(XLS)

Table S3 **GO-Term definitions of microarray data.**
(XLS)

Table S4 **GO-Term definitions of T877A-AR Gene-sets 1–10.**
(XLS)
