# Supplementary material for: Proteomic-Coupled-Network Analysis of T877A-Androgen Receptor Interactomes Can Predict Clinical Prostate Cancer Outcomes between White (Non-Hispanic) and African-American Groups
Source: PLoS One. 2014 Nov 19;9(11):e113190. doi: 10.1371/journal.pone.0113190 (PMC4237393; doi:10.1371/journal.pone.0113190)
Supplement: Figure S1 — CaP 10-year survival outcomes forT877A-AR Gene-sets 3–10. (PDF) [file pone.0113190.s001.pdf]

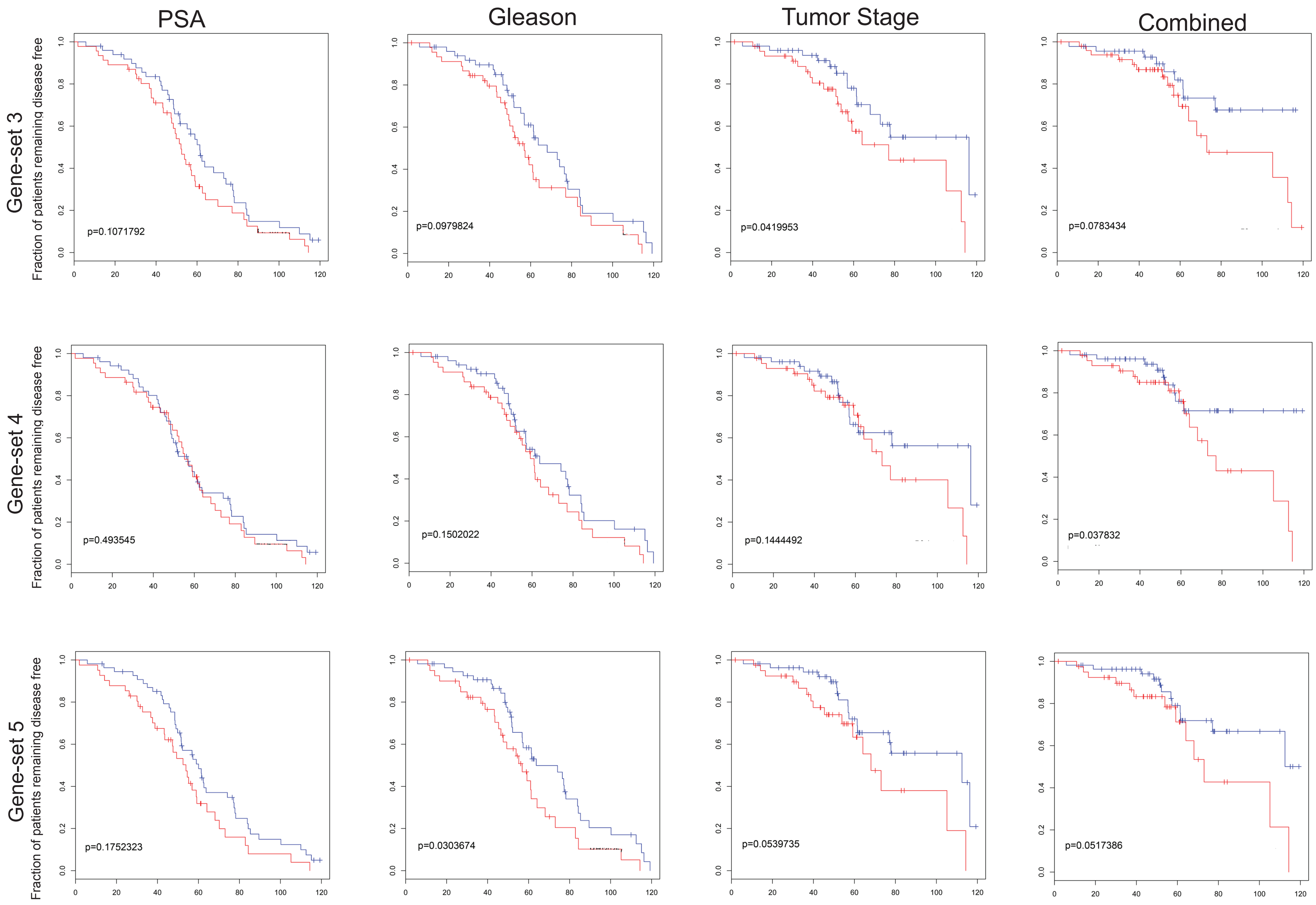
PSA
Gleason
Tumor Stage
Combined
Gene-set 3
Gene-set 4
Gene-set 5
Fraction of patients remaining disease free
p=0.1071792
p=0.0979824
p=0.0419953
p=0.0783434
p=0.493545
p=0.1502022
p=0.1444492
p=0.037832
p=0.1752323
p=0.0303674
p=0.0539735
p=0.0517386

Figure S1

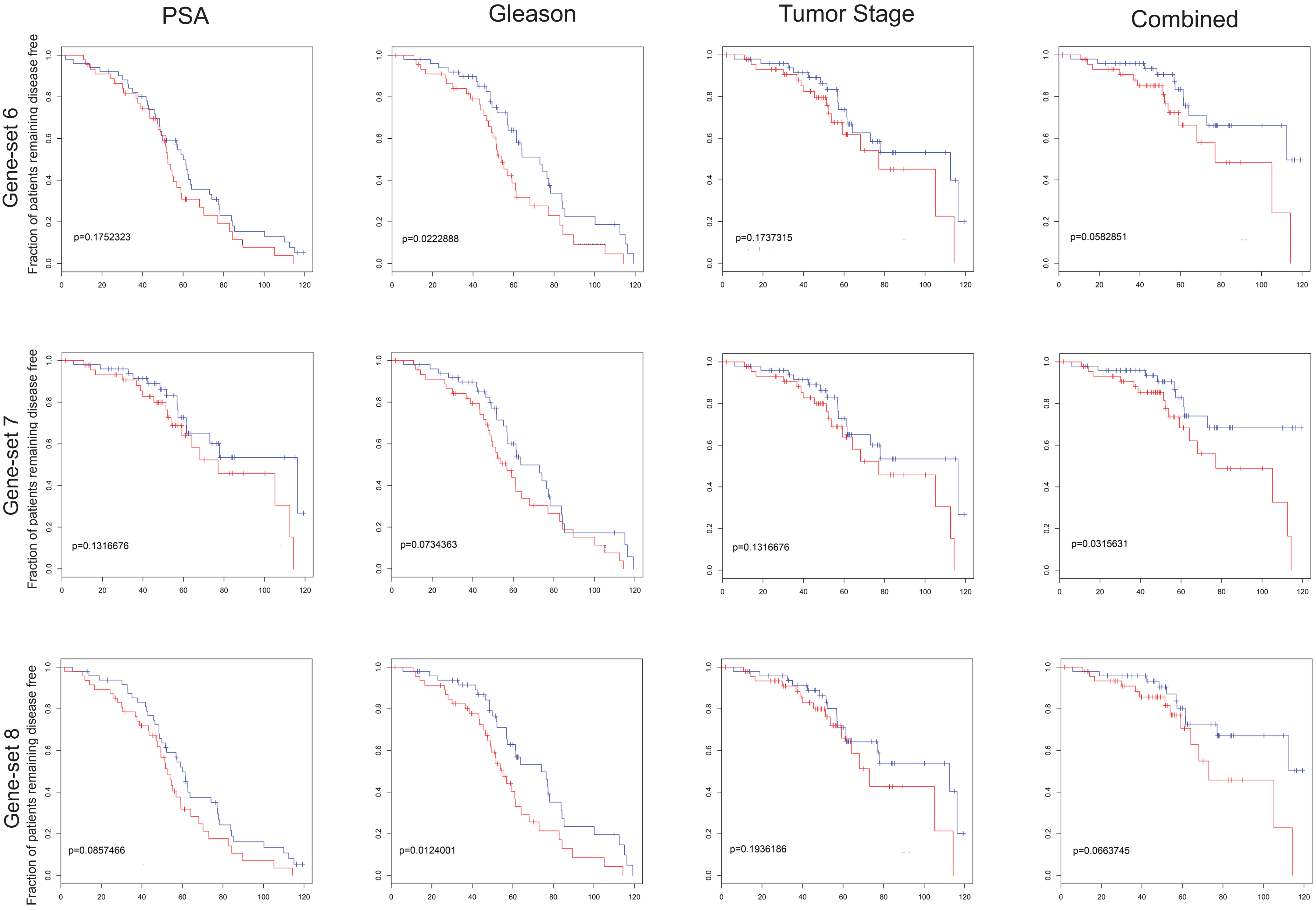
PSA
Gleason
Tumor Stage
Combined
Gene-set 6
Gene-set 7
Gene-set 8
Fraction of patients remaining disease free
p=0.1752323
p=0.0222888
p=0.1737315
p=0.0582851
p=0.1316676
p=0.0734363
p=0.1316676
p=0.0315631
p=0.0857466
p=0.0124001
p=0.1936186
p=0.0663745

Figure S1

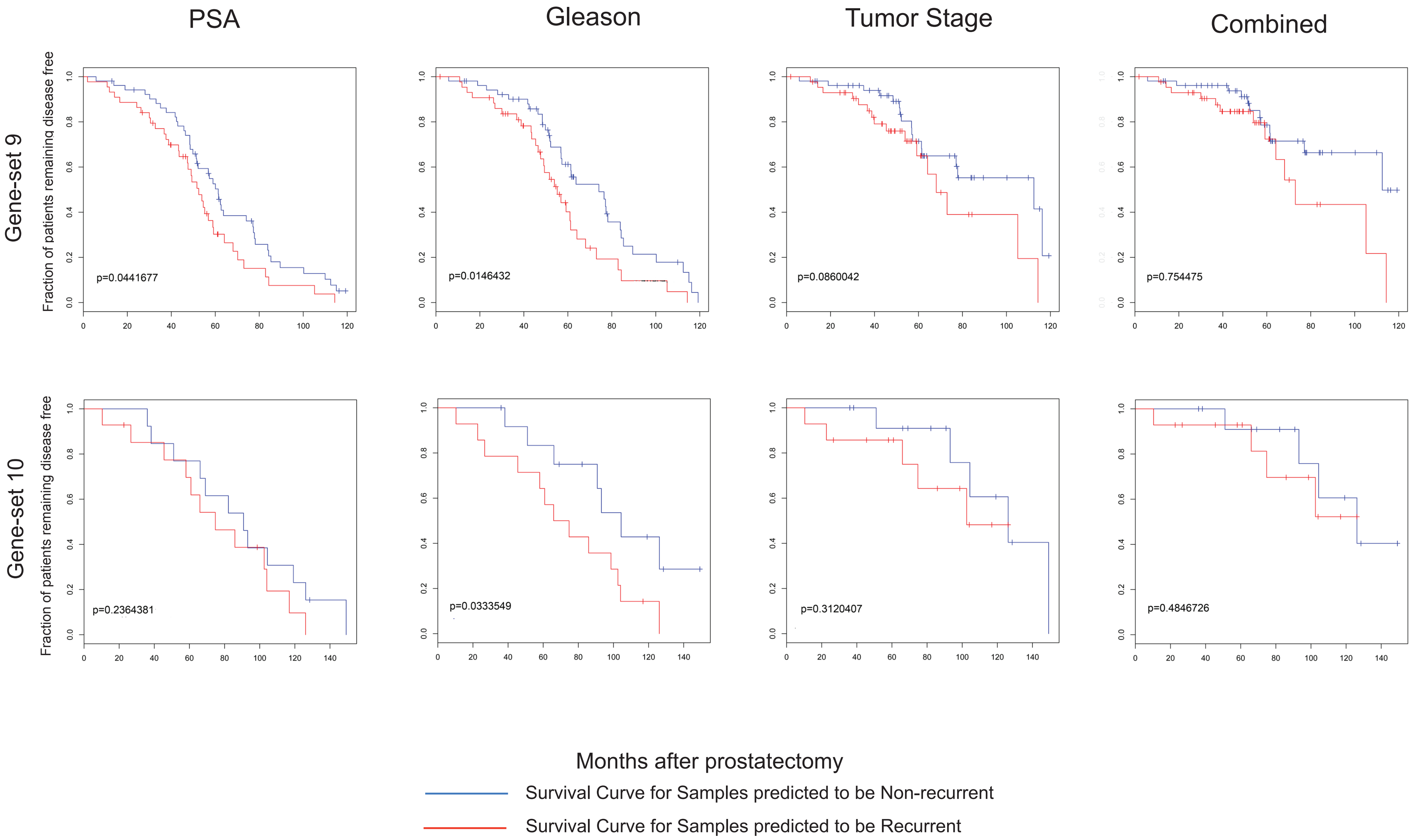
PSA
Gleason
Tumor Stage
Combined
Gene-set 9
Gene-set 10
Fraction of patients remaining disease free
p=0.0441677
p=0.0146432
p=0.0860042
p=0.754475
p=0.2364381
p=0.0333549
p=0.3120407
p=0.4846726
Months after prostatectomy
Survival Curve for Samples predicted to be Non-recurrent
Survival Curve for Samples predicted to be Recurrent

Figure S1
