# Supplementary material for: Proteomic-Coupled-Network Analysis of T877A-Androgen Receptor Interactomes Can Predict Clinical Prostate Cancer Outcomes between White (Non-Hispanic) and African-American Groups
Source: PLoS One. 2014 Nov 19;9(11):e113190. doi: 10.1371/journal.pone.0113190 (PMC4237393; doi:10.1371/journal.pone.0113190)
Supplement: Figure S2 — Disease-free survival outcomes of 4 non-CaP cancers for Gene-sets 3–10. (PDF) [file pone.0113190.s002.pdf]

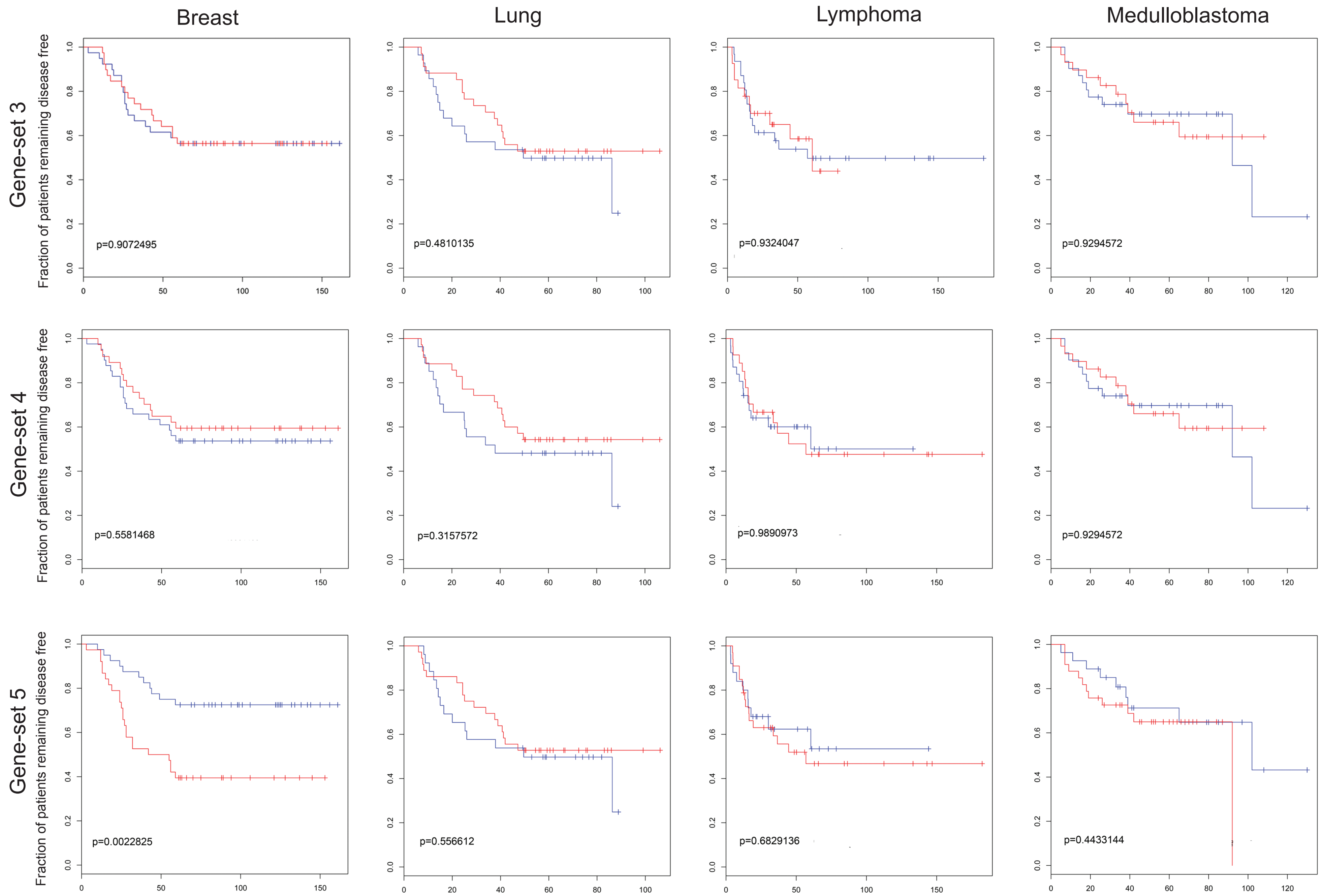

Breast
Lung
Lymphoma
Medulloblastoma
Gene-set 3
Gene-set 4
Gene-set 5
Fraction of patients remaining disease free
p=0.9072495
p=0.4810135
p=0.9324047
p=0.9294572
p=0.5581468
p=0.3157572
p=0.9890973
p=0.9294572
p=0.0022825
p=0.556612
p=0.6829136
p=0.4433144

Figure S2

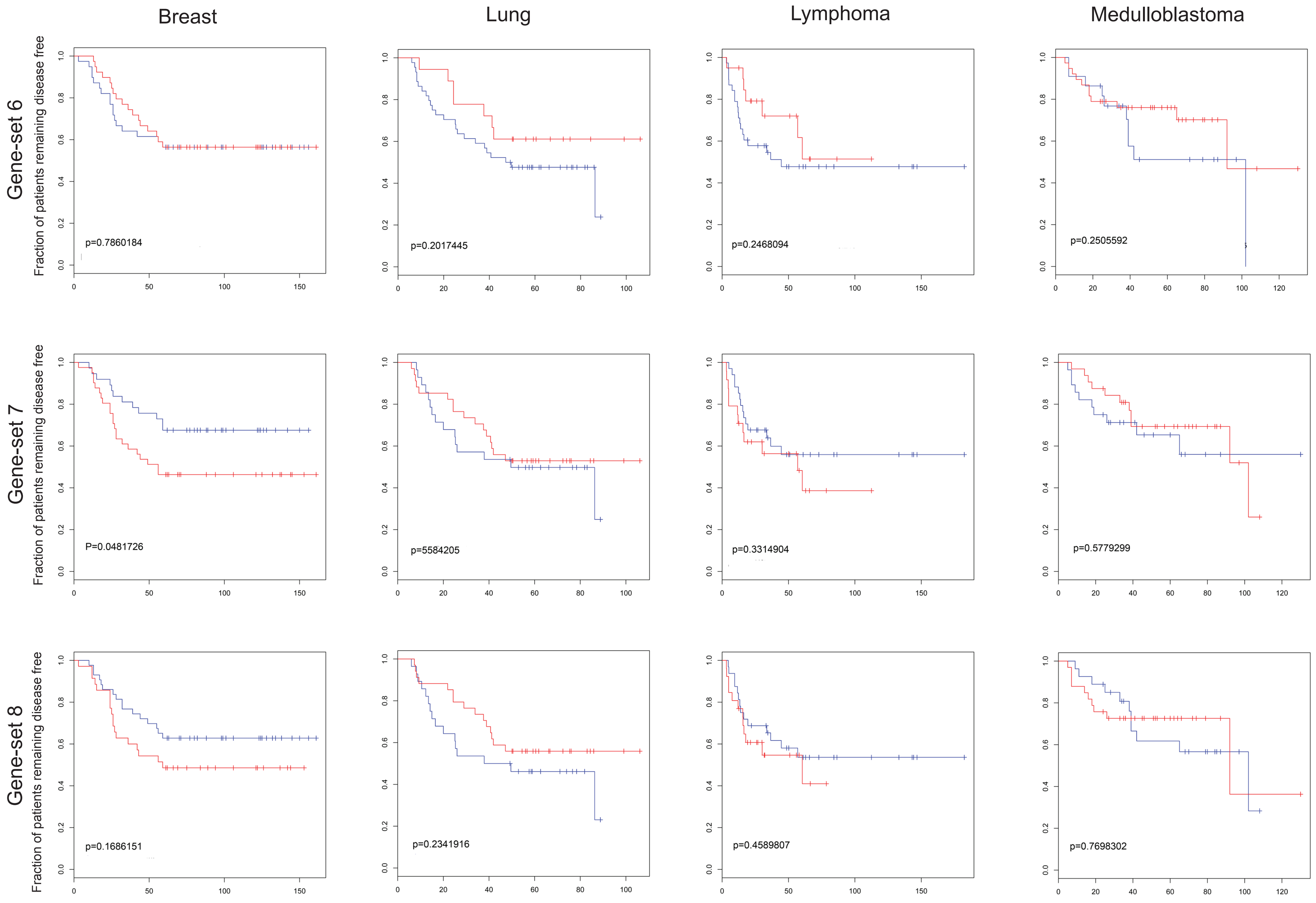

Breast
Lung
Lymphoma
Medulloblastoma
Gene-set 6
Gene-set 7
Gene-set 8
Fraction of patients remaining disease free
p=0.7860184
p=0.2017445
p=0.2468094
p=0.2505592
P=0.0481726
p=5584205
p=0.3314904
p=0.5779299
p=0.1686151
p=0.2341916
p=0.4589807
p=0.7698302

Figure S2

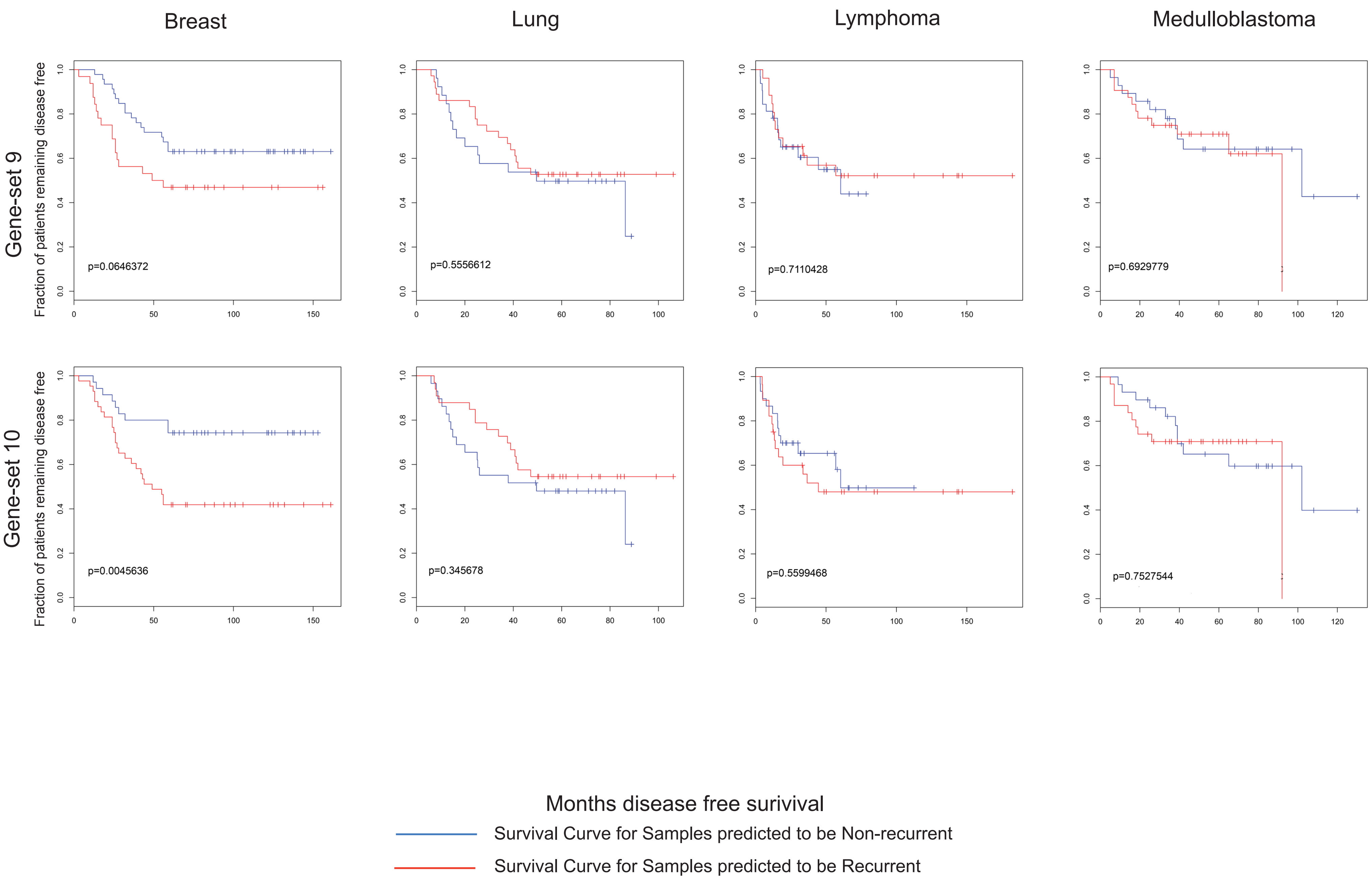
Breast
Lung
Lymphoma
Medulloblastoma
Gene-set 9
Gene-set 10
Fraction of patients remaining disease free
p=0.0646372
p=0.5556612
p=0.7110428
p=0.6929779
p=0.0045636
p=0.345678
p=0.5599468
p=0.7527544
Months disease free surivival
Survival Curve for Samples predicted to be Non-recurrent
Survival Curve for Samples predicted to be Recurrent

Figure S2
